# Evaluation of Sexual Dimorphism Using Condylar and Coronoid Mandibular Parameters in Orthopantomograms: A Pilot Study

**DOI:** 10.7759/cureus.62367

**Published:** 2024-06-14

**Authors:** Abirami Arthanari, Shanmathy Sureshbabu, Karthikeyan Ramalingam, Vignesh Ravindran, Lavanya Prathap

**Affiliations:** 1 Department of Forensic Odontology, Saveetha Dental College and Hospitals, Saveetha Institute of Medical and Technical Sciences, Saveetha University, Chennai, IND; 2 Department of Oral Pathology and Microbiology, Saveetha Dental College and Hospitals, Saveetha Institute of Medical and Technical Sciences, Saveetha University, Chennai, IND; 3 Department of Pedodontics and Preventive Dentistry, Saveetha Dental College and Hospitals, Saveetha Institute of Medical and Technical Sciences, Saveetha University, Chennai, IND; 4 Department of Anatomy, Saveetha Medical College and Hospitals, Saveetha Institute of Medical and Technical Sciences, Saveetha University, Chennai, IND

**Keywords:** condylar ramus height, coronoid ramus height, sexual dimorphism, digital orthopantomogram, human identification, forensic identification, mandibular metrics, mandibular features, gender assessment, sex determination

## Abstract

Background

Gender determination is critical to forensic science and medico-legal applications. Given that it is the most dimorphic bone in the skull and is frequently found intact, the mandibular bone may be extremely important in determining gender. Orthopantomograms (OPGs) are quite helpful in accurately estimating age and sex in this regard. It is a laborious task for forensics to determine the gender of victims of mass casualties, natural disasters, and severely dismembered bodies. The mandible, which is susceptible to development spurts, has a high degree of accuracy for determining sex.

Aim

This study aims to evaluate the potential use of coronoid height and condylar height as reliable anatomical markers for determining gender.

Materials and methods

In this study, 100 samples were used as study samples, 50 of which were male and 50 of which were female, in the age group of 20-30 years. The OPGs were obtained using a Planmeca Promax Scara 3 Digital OPG Machine (Planmeca, Helsinki, Finland), with settings of 70 kVp, 8 mA for 0.9 seconds, ensuring a 1:1 ratio. The images were then transferred to Planmeca Romexis® Viewer Software, Version 6.0 (Planmeca Oy, Helsinki, Finland) for measurement recording.

Results

Descriptive statistical analysis was done for this study and discriminant analysis was also done to create a population-specific formula. Results showed that the standard mean error for males concerning condylar height was 2.3 and coronoid height was 0.7. The standard mean error for females by condylar height was 1.6 and coronoid height was 0.6. The p-value was significant for coronoid height in both males and females. The p-value was not clinically significant for condylar height in both males and females.

Conclusion

The study's findings indicate that a larger mandibular angle is advantageous for gender assessment and helps with gender dimorphism. Out of both the parameters evaluated, coronoid height has shown statistical significance in both males and females. Hence, the study concludes that the parameter, coronoid height can be utilized to assess the gender of an individual.

## Introduction

Gender estimation is of tremendous importance to forensic odontology since it provides a lot of information for identifying unknown individuals. New approaches are continuously being investigated to improve the precision and dependability of gender estimation with developing technology. An important area of forensic anthropology is sexual dimorphism using mandibular characteristics, which plays a major role in accurately determining the gender of an individual. Males and females differ morphologically in the mandible, a skeletal component that is sexually dimorphic [1]. Analyzing coronoid and condylar height is one such new approach that has the potential to provide perceptive data. The coronoid and condylar processes, significant parts of the mandible, are essential for the smooth functioning of the temporomandibular joint (TMJ). Orthopantomograms (OPGs) offer a reliable and accurate method for measuring the heights of the condylar and coronoid rami due to their comprehensive panoramic view, consistent imaging technique, and ability to clearly visualize and measure critical anatomical landmarks. Their role in clinical diagnosis, treatment planning, and follow-up makes them an indispensable tool in dental and maxillofacial radiology. Even though these structures are mostly related to how the jaw functions, they can also be accurate markers of sexual dimorphism [2]. Sexual dimorphism is the physiological differences between males and females that help forensic investigators identify the gender of unidentified people by taking advantage of these disparities [2,3].

Dental features including tooth size, dental morphology, and the examination of dental remains for identification purposes have played an important role in the field of Forensics. However, in the pursuit of more precise and sophisticated gender assessment techniques, researchers have turned their attention to other anatomical characteristics, such as those connected to the mandible [4]. It is one of the sexually dimorphic bones exhibiting distinct differences between males and females. The two processes of the mandible, condyle and coronoid are found to show varied heights in males and females [4,5]. In forensic and anthropological studies, the measurement of condylar ramus height and coronoid ramus height using OPGs can be valuable for sex determination. The coronoid height can be simply termed as the distance that extends between the highest point in the coronoid process and the inferior border of the mandible. The condylar ramus height tends to be greater in males compared to females. This difference is attributed to the generally larger and more robust mandibles in males. Similarly, condylar height is the distance between the highest point in the condylar process and the inferior border of the mandible [6]. Similar to the condylar ramus height, the coronoid ramus height is typically greater in males. The larger coronoid process in males is associated with stronger masticatory muscles and overall bone density. According to previous research, there is sexual dimorphism evident in these measurements, with males typically having greater coronoid and condylar heights than females. These mandibular components vary in size and shape due to the influence of sex hormones on bone development, resulting in dimorphism [7].

Using coronoid and condylar height for gender assessment has several advantages. Primarily, these measurements are invaluable in forensic scenarios, where non-invasive techniques are preferred, as they can be obtained from skeletal remains without causing damage. Numerous studies among various populations have demonstrated the accuracy and reliability of these measurements, validating their use across different ethnic groups [8]. Ongoing research aims to refine and validate methods for determining gender based on mandibular parameters. Geometric morphometrics and three-dimensional (3D) surface scanning are powerful tools that significantly enhance the precision and accuracy of measurements in various fields, including biology, anthropology, paleontology, and archaeology. So, geometric morphometrics and 3D surface scanning together provide a sophisticated framework for morphological studies, enhancing both the precision and accuracy of measurements and analyses. Further, an interdisciplinary approach among radiologists, computer scientists, and anthropologists has led to the development of automated quantification algorithms, making the analysis process more efficient and reducing subjectivity [9].

The mandible is frequently better preserved than other traditional techniques that depend on the presence of particular teeth, which may be absent or damaged in forensic situations. The mandible is the second best marker of gender determination after the pelvis, as the mandible is sexually diversified. Furthermore, the mandible is a good sign of sexual dimorphism even in older skeletal remains, as it displays this trait throughout the life of an individual. More research is needed to use coronoid and condylar height analysis in forensic settings through in-depth and population-specific investigations [10]. To guarantee the accuracy and dependability of the gender estimation results, factors including age, population variability, and possible hormonal influences must be taken into account. These mandibular parameters may become essential elements of forensic investigations as technology advances and our knowledge of bone anatomy expands, providing a useful tool for identifying and bringing closure to instances involving unidentified individuals. By comparing the condylar and coronoid ramus heights of an unknown subject with the established baselines, forensic experts can infer the sex of the individual [3]. The present study is undertaken to assess the rationale of the mandibular parameters, coronoid, and condylar height in sex estimation.

## Materials and methods

**Figure 1:**
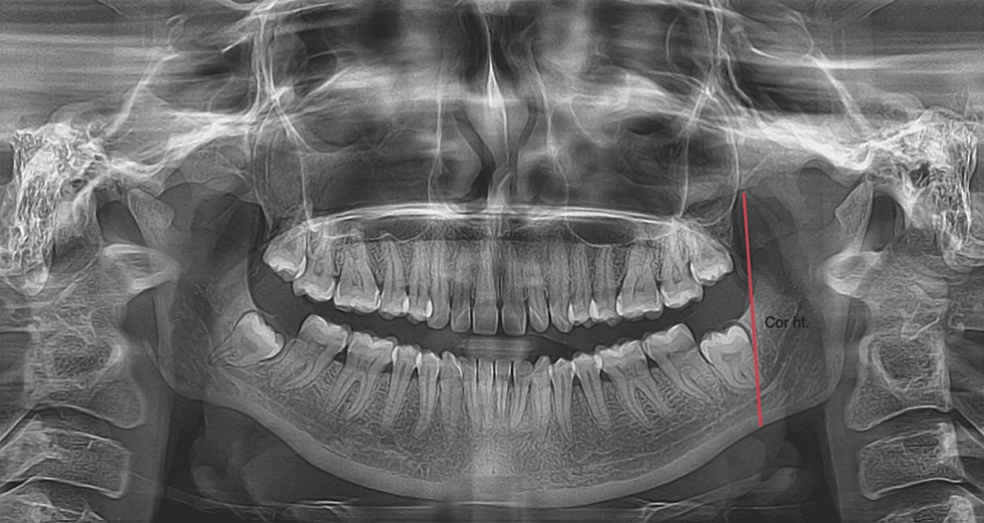
An orthopantomogram represents the coronoid ramus height (Cor. ht) of the mandible

This study was employed to establish a population-specific criterion for evaluating sex and age with mandibular parameters as a measure. For this study, we collected digital OPGs from the archives of the Department of Oral Medicine and Radiology at Saveetha Dental College and Hospitals, Chennai, India. The sample size was calculated using G*Power software (version 3.1.9.4; Heinrich Heine Universität Düsseldorf, Düsseldorf, Germany) to ensure a statistical power of 95% with a significance level (alpha error probability) of 0.05. The calculated sample size was 92, and we included a total of 100 samples to ensure adequate statistical power. To prevent bias, an equal number of samples were collected from males and females, with 50 samples each. The age range included in the study was 20-30 years. The parameters evaluated were condylar ramus height and coronoid ramus height. These parameters were assessed to decide whether they can contribute to age and sex determination. The study included OPG radiographs that were visible, well-aligned, and had known sex information, while those with artifacts or pathological conditions were excluded. Measurements were taken using Planmeca Romexis® Viewer, Version 6.0 (Planmeca Oy, Helsinki, Finland). Statistical analysis was performed using SPSS for Windows, Version 16 (Released 2007; SPSS Inc., Chicago, IL, USA). The study received approval from the Institutional Human Ethics Committee of Saveetha Dental College (IHEC/SDC/FACULTY/22/FO/059). The coronoid ramus height of the mandible measures the distance between the highest point in the coronoid process and the inferior border of the mandible shown in Figure 1.

**Figure 2:**
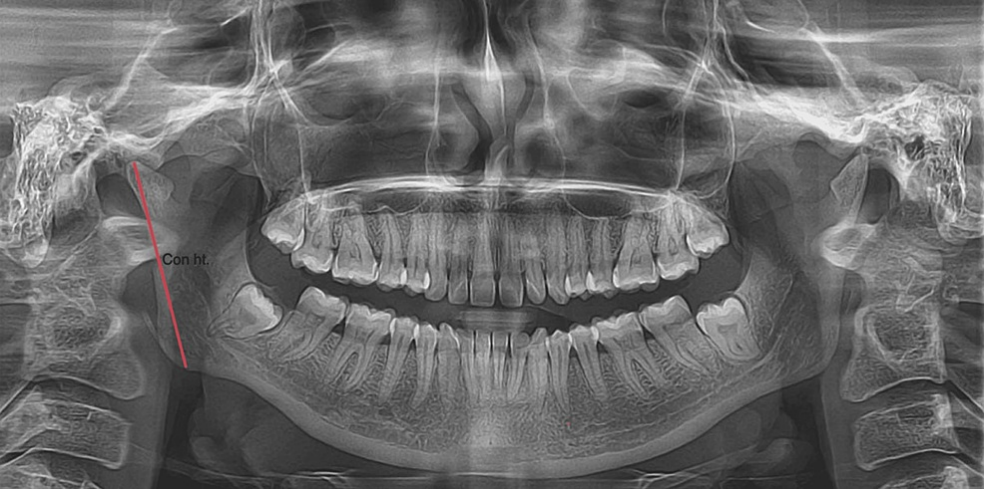
An orthopantomogram represents the condylar ramus height (Con. ht) of the mandible

The condylar ramus height of the mandible shows the distance between the highest point in the condylar process and the inferior border of the mandible represented in Figure 2.

## Results

The results of this study show that there are significant differences in the mandibular angle between both genders. The analysis of mandibular parameters like condylar ramus height and coronoid ramus height of males and females are mentioned below. In males, mandibular parameters show a standard deviation of 16.3 for condylar ramus height and 5.2 for coronoid ramus height. Also in males, the parameter coronoid ramus height has a standard error mean of 0.7, whereas condylar ramus height has a 2.3 standard error mean. The p-value is 0.00 in coronoid ramus height was statistically significant in males and the p-value of condylar ramus height is 0.37, which is statistically not significant. The means of all the parameters on their combined means within the category (20-30 years) of males were illustrated in Table 1.

**Table 1:** Combined analysis of condylar and coronoid height for males In males, the coronoid ramus height p-value of 0.00 was statistically significant, while the condylar ramus height p-value of 0.37 was statistically not significant * p-value is statistically significant

Parameter (male)	N	Mean	Standard deviation	Standard error mean	Sig. p-value
Condylar ramus height	50	60.9	16.3	2.3	0.37
Coronoid ramus height	50	69	5.2	0.7	0.00*

In females, mandibular parameters show a standard deviation of 1.6 for condylar ramus height and 0.6 for coronoid ramus height. Also, coronoid ramus height has a standard error mean of 4.5, whereas condylar ramus height has an 11.8 standard error mean. The p-value is 0.00 in coronoid ramus height was statistically significant in females and the p-value of condylar ramus height is 0.37 which is statistically not significant. The means of all the parameters on their combined means within the category (20-30 years) of females were illustrated in Table 2.

**Table 2:** Combined analysis of condylar and coronoid height for females In females, the p-value for coronoid ramus height was found to be statistically significant at 0.00, whereas the p-value for condylar ramus height was found to be statistically not significant at 0.37 * p-value is statistically significant

Parameter (female)	N	Mean	Standard deviation	Standard error mean	Sig. p-value
Condylar ramus height	50	61.5	11.8	1.6	0.37
Coronoid ramus height	50	64.3	4.5	0.6	0.00*

In the discriminant function analysis, condylar ramus height did not contribute towards sex determination and was consequently not utilized in deriving the formula. From Table 2, it can be noted that the standardized coefficient is greater in magnitude for coronoid ramus height (0.101, which reaches 1). Thus, coronoid ramus height will have the greatest impact on sex determination. The discriminant analysis equation obtained from the data to determine sex is given in Table 3.

**Table 3:** Regression analysis to estimate the gender of coronoid ramus height From these values, the following formula could be derived: Gender = -21.725 + 0.101 × coronoid ramus height

Mandibular parameter	Function
		Coronoid ramus height	0.101
Constant	-21.725

The predicted group membership is based on gender and for males, 41 out of 50 cases were correctly classified, which is 82%. For females, 49 out of 50 cases were correctly classified, which is 98%. Overall, 90 out of 100 cases were correctly classified, which is 90%. This means that the model correctly predicted the gender for 90% of the cases in the original group which is represented in Table 4.

**Table 4:** Prediction analysis of sex estimation using condylar ramus height and coronoid ramus height In this prediction analysis, 90% of the original group cases were correctly classified

Gender	Predicted sex		Total (N)
	Female (N)	Male (N)	
Female	49 (98%)	1 (2%)	50
Male	9 (18%)	41 (82%)	50

## Discussion

Analyzing mandibular characteristics, such as coronoid and condylar height, can reveal gender. The anterior bony projection is referred to as the coronoid process, and the rounded protrusion at the back of the jaw is called the condyle. The morphological differences between the male and female coronoid processes and condyles give rise to these distinctive features of the mandible. Measurements of coronoid and condylar heights are used to determine gender since they are regarded as reliable markers [6].

In this study, the mandibular characteristics in both males and females were evaluated. In males, the standard deviation for condylar ramus height was 16.3, whereas for coronoid ramus height, it was 5.2. Furthermore, the standard error mean for coronoid ramus height was 0.7, whereas condylar ramus height was 2.3. The p-value for coronoid ramus height was 0.00, indicating statistical significance, whereas for condylar ramus height, it was 0.37, indicating no statistical significance. In females, the standard deviation for condylar ramus height was 1.6, and for coronoid ramus height, it was 0.6. The standard error mean for coronoid ramus height was 4.5, and for condylar ramus height, it was 11.8. The p-value for coronoid ramus height was 0.00, statistically significant, while for condylar ramus height, it was 0.37, not significant. The predictive model based on gender correctly classified 82% of males and 98% of females, with an overall correct classification rate of 90%, indicating the model's effectiveness in predicting gender based on mandibular parameters.

In the Kaur et al. study, there was a significant difference found in males and females in maximum ramus width, minimum ramus width, condylar height, and coronoid height and between males and females in condylar height and coronoid height. Furthermore, there was a statistically significant difference between males and females in coronoid height [7]. Similarly, in our study, there was a statistical significance of 0.00 in coronoid ramus height was found with a standard deviation of 5.2+/-0.7 in males.

Determining the gender of an individual, an aspect of anthropology relies on examining certain skeletal characteristics that show differences between males and females. There are bones in the skeleton that provide valuable information for identifying the gender of a person, helping in understanding their biological traits and aiding in the identification process [11]. The pelvis is often considered a reliable bone when it comes to gender estimation. Features like the inlet, notch, subpubic angle, and pelvic shape differ significantly between males and females [12]. The skull is another area of the body, where dimorphic features can be observed. Certain characteristics, such as an eyebrow ridge, mastoid process, and mandibular ramus, show variations between males and females. Forensic experts utilize these features and often employ techniques like geometric morphometrics and advanced imaging for more accurate analysis [13].

Long bones like the femur (thigh bone) and humerus (upper arm bone) also play a role in estimating gender. These bones undergo changes in size and proportions that differ between males and females. While not as reliable as cranial indicators, long bones provide additional information, for a more comprehensive assessment. In general, determining gender based on bones requires an approach that takes into account skeletal components and utilizes both macroscopic and microscopic analyses [14]. The continuous advancements in technology have greatly improved the precision of these evaluations, which play a role in the field's endeavors to ensure comprehensive and dependable forensic identifications [15,16]. Forensic specialists can determine the gender of their skeletal remains by measuring certain characteristics and recognizing these differences. A significant location to evaluate sexual dimorphism is the mandibular ramus, a striking feature of the mandible. Ramus height, length, and width measurements offer important information regarding one’s gender [17]. When compared to females, men usually exhibit a bigger and more sturdy ramus. The overall variations in strength and size between the sexes are reflected in this dimorphism. Another area where sexual dimorphism is noticeable is the mandibular corpus, or the horizontal body of the jaw [18].

The difference between the mandibles of men and women can be partly explained by measurements of the height and length of the corpus. Men often have a larger and stronger mandibular corpus than females, which highlights the significance of this measure in estimating gender [19]. One characteristic that contributes to sexual dimorphism is the gonial angle, which is produced at the intersection of the ramus and the corpus. Studies reveal that men frequently have a larger gonial angle than women [20]. This anatomical distinction is important in determining gender, even though there might be differences in various populations. There are many sexually dimorphic traits in the symphysis, which is the point where the left and right halves of the mandible converge at the midline [21]. When estimating gender, the form of the symphysis and the existence or lack of a mental eminence are important factors. Generally speaking, ladies have a more pointed and graceful appearance, whereas males have a square-shaped symphysis [22,23].

Mandibular sexual dimorphism is not only an anatomical curiosity, but it also has important forensic ramifications. In cases where additional gender markers are absent from skeletal remains, the examination of mandibular features becomes essential for creating an individual's biological profile [12]. In forensic anthropology, accurate gender estimates are based on the unique physical differences between male and female mandibles. Although mandibular sexual dimorphism is a useful tool, there are a few things to be aware of [22]. Complexities in the study may arise due to population-specific variances and the possible influence of environmental factors. Furthermore, age-related alterations may have an impact on mandibular morphology, highlighting the necessity of a thorough and nuanced method for determining gender. The accuracy and consistency of mandibular parameter assessments of sexual dimorphism are being improved by technological innovations like geometric morphometrics and 3D imaging. By offering more thorough and precise evaluations, these methods aid in the continuous improvement of forensic procedures [23].

Future studies could benefit from interdisciplinary collaboration with geneticists and other forensic specialists. Gender diagnoses that are more reliable and accurate may result from the integration of statistical models and genetic markers with mandibular parameter assessments [7]. These cooperative initiatives guarantee a comprehensive approach to forensic anthropology by fusing state-of-the-art technologies with conventional morphological analysis [19]. The foundation of gender estimation in forensic anthropology is sexual dimorphism using mandibular parameters. Based only on skeletal remains, the physical distinctions between male and female mandibles reveal important information about an individual's likely gender [24].

When compared to females, males often have higher coronoid and condylar heights. Males have larger dimensions because their skeletons are larger overall. Difficulties like individual and population-specific variances need to be taken into account when interpreting these measures. The improvement of sexual dimorphism evaluations in coronoid and condylar heights is made possible by interdisciplinary collaboration and technological breakthroughs like 3D imaging [25].

The accuracy and reliability of sexual dimorphism assessments in mandibular parameters will be a key factor in the success of forensic investigations going forward, ultimately helping with the reconstruction of life histories and identification of individuals as technology and collaborative research continue to progress [10].

Limitations

Evaluating sexual dimorphism in condylar and coronoid measurements using OPG has several limitations. Measurement accuracy can be affected by imaging distortions, and anatomical variability among individuals can complicate identifying consistent sexual dimorphism patterns. Operator-dependent inconsistencies in positioning and measurement techniques further impact result reliability. The 2D nature of OPG can lead to inaccuracies in representing 3D structures, making precise measurements difficult. Additionally, overlapping anatomical structures can obscure critical details, reducing diagnostic clarity and precision needed for accurately determining sex differences. These limitations should be considered in future research.

## Conclusions

There is a statistical difference between coronoid and condylar height in males and females. Gender can be estimated using the given mandibular parameter, coronoid height. In conclusion, it has been demonstrated that using OPG imaging to ascertain gender based on mandibular parameters is a practical and flexible technique in several domains, including dentistry, forensic anthropology, and clinical diagnostics. The mandible, a prominent facial bone, is a strong indication of age and gender since it changes regularly over an individual's lifetime. The possibility of gender prediction using mandibular traits can be advantageous in forensic scenarios. Forensic anthropologists employ OPG scans to establish the age at death of unidentified skeletal remains. Interdisciplinary research collaborations and continuous advancements in imaging technology could significantly enhance the precision and reliability of age and gender estimate methodologies.

## References

[1] Akhlaghi M, Khalighi Z, Vasigh S, Yousefinejad V (2014). Sex determination using mandibular anthropometric parameters in subadult Iranian samples. J Forensic Leg Med.

[2] Suzuki K, Nakano H, Inoue K (2020). Examination of new parameters for sex determination of mandible using Japanese computer tomography data. Dentomaxillofac Radiol.

[3] Vinay G, Gowri SRM, Anbalagan J (2013). Sex determination of human mandible using metrical parameters. J Clin Diagn Res.

[4] Trivunov N, Petrović B, Milutinović S (2022). Sex and age determination of human mandible using anthropological parameters and TCI and Kvaal methods: study of a Serbian medieval sample. Surg Radiol Anat.

[5] Ulusoy AT, Ozkara E (2022). Radiographic evaluation of the mandible to predict age and sex in subadults. Acta Odontol Scand.

[6] Shakya T, Maharjan A, Pradhan L (2022). Morphometric analysis of mandibular ramus for sex determination on orthopantomogram. J Nepal Health Res Counc.

[7] Kaur R, Pallagatti S, Aggarwal A, Mittal PG, Singh M, Patel ML (2021). Mandibular ramus as a strong expressor of sex determinations: a digital radiographic study. J Pharm Bioallied Sci.

[8] Samatha K, Byahatti SM, Ammanagi RA, Tantradi P, Sarang CK, Shivpuje P (2016). Sex determination by mandibular ramus: a digital orthopantomographic study. J Forensic Dent Sci.

[9] Verma P, Mahajan P, Puri A, Kaur S, Mehta S (2020). Gender determination by morphometric analysis of mandibular ramus in sriganganagar population: a digital panoramic study. Indian J Dent Res.

[10] Saini V, Srivastava R, Rai RK, Shamal SN, Singh TB, Tripathi SK (2011). Mandibular ramus: an indicator for sex in fragmentary mandible. J Forensic Sci.

[11] Okkesim A, Sezen Erhamza T (2020). Assessment of mandibular ramus for sex determination: retrospective study. J Oral Biol Craniofac Res.

[12] Lin C, Jiao B, Liu S, Guan F, Chung NE, Han SH, Lee UY (2014). Sex determination from the mandibular ramus flexure of Koreans by discrimination function analysis using three-dimensional mandible models. Forensic Sci Int.

[13] More CB, Vijayvargiya R, Saha N (2017). Morphometric analysis of mandibular ramus for sex determination on digital orthopantomogram. J Forensic Dent Sci.

[14] Kadkhodazadeh M, Shafizadeh M, Rahmatian M, Safi Y, Amid R (2022). Determination of the volume and density of mandibular ramus as a donor site using CBCT. J Maxillofac Oral Surg.

[15] Shree B, Soni S, Sharma SK, Handge K, Kumar A, Das SS, Puri N (2023). Analytical study of mandible: prerequisite for sex determination. J Pharm Bioallied Sci.

[16] Del Bove A, Menéndez L, Manzi G, Moggi-Cecchi J, Lorenzo C, Profico A (2023). Mapping sexual dimorphism signal in the human cranium. Sci Rep.

[17] Shah PH, Venkatesh R, More CB, Vaishnavee V (2020). Age- and sex-related mandibular dimensional changes: a radiomorphometric analysis on panoramic radiographs. Indian J Dent Res.

[18] Ingaleshwar P, Bhosale S, Nimbulkar G, Britto F, Chandrappa PR, Hosur MB (2023). Mandibular ramus - an indicator for gender determination: a digital panoramic study in Bagalkot population. J Oral Maxillofac Pathol.

[19] Kano T, Oritani S, Michiue T (2015). Postmortem CT morphometry with a proposal of novel parameters for sex discrimination of the mandible using Japanese adult data. Leg Med (Tokyo).

[20] Ishwarkumar S, Pillay P, Haffajee MR, Satyapal KS (2017). Morphometric analysis of the mandible in the Durban Metropolitan population of South Africa. Folia Morphol (Warsz).

[21] Alias A, Ibrahim A, Abu Bakar SN (2018). Anthropometric analysis of mandible: an important step for sex determination. Clin Ter.

[22] Koju S, Maharjan N, Rajak RR, Yadav DK, Bajracharya D, Ojha B (2021). Assessment of sexual dimorphism in mandibular ramus: an orthopanoramic study. Kathmandu Univ Med J.

[23] Arthanari A, Dogalli N, Vidhya A, Rudraswamy S (2020). Age estimation from second & third molar by modified gleiser and hunt method: a retrospective study. Indian J Forensic Med Toxicol.

[24] Mączka G, Kulus M, Grzelak J, Porwolik M, Dobrzyński M, Dąbrowski P (2022). Morphology of the antegonial notch and its utility in the determination of sex on skeletal materials. J Anat.

[25] Sairam V, Geethamalika MV, Kumar PB, Naresh G, Raju GP (2016). Determination of sexual dimorphism in humans by measurements of mandible on digital panoramic radiograph. Contemp Clin Dent.

